# Effects of Safflower Yellow on the Treatment of Severe Sepsis and Septic Shock: A Randomized Controlled Clinical Trial

**DOI:** 10.1155/2016/3948795

**Published:** 2016-02-18

**Authors:** Xiao-jin Li, Ru-rong Wang, Yan Kang, Jin Liu, Yun-xia Zuo, Xue-feng Zeng, Gong Cheng

**Affiliations:** ^1^Department of Anesthesiology, West China Hospital of Sichuan University, Chengdu 610041, China; ^2^Department of Intensive Care Unit, West China Hospital of Sichuan University, Chengdu 610041, China; ^3^Department of Respiratory Medicine, The People's Hospital of Pujiang County, Sichuan 611630, China; ^4^Department of Nephrology, The People's Hospital of Pujiang County, Sichuan 611630, China

## Abstract

*Objective.* To evaluate the clinical effect of safflower yellow on the treatment of severe sepsis and septic shock.* Methods.* 85 patients with severe sepsis and septic shock were randomly selected to receive either therapy according to the international guidelines for management of severe sepsis and septic shock (Surviving Sepsis Campaign 2012) (control group,* n* = 45) or conventional therapy plus safflower yellow (study group,* n* = 40). The 28-day mortality and 28-day Kaplan-Meier survival curves were compared as primary outcomes.* Results.* The 28-day mortality from all causes and in-hospital mortality were significantly lower in the study group (50%, 17.5%) as compared to the control group (78.58%, 54.76%) (*P* = 0.007, all causes,* P* < 0.001, in-hospital), and the 28-day Kaplan-Meier survival curve was higher in the study group than in the control group (*P* = 0.008, all causes,* P* < 0.001, in-hospital, Log Rank). 72 hours after treatment, secondary outcomes including heart rate, leukocyte counts, lactate levels, and platelet counts of patients in the study group were ameliorated significantly as compared with the control group.* Conclusion.* This study offers a potential new strategy employing safflower yellow to more effectively treat patients with severe sepsis and septic shock. This trial is registered with identifier ChiCTR-TRC-14005196.

## 1. Introduction

Severe sepsis and septic shock are common in critical care medicine and are usually associated with high mortality. There are an estimated 751,000 cases (3.0 cases per population of 1000 persons) of sepsis or septic shock in the United States each year [1] and even very high morbidity of 7.68% in Netherlands [2], which is accompanied by high mortality in many clinical trials [3–5]. The rapid progression, poor overall prognosis, and high mortality of severe sepsis and septic shock have stimulated many researchers and intensivists in critical care medicine to search for better means of treatment. However, improvement of outcome is still a complex issue, and no “magic bullet” has to date been found. The Surviving Sepsis Campaign (SSC) which offers international guidelines for management of severe sepsis and septic shock has been updated in the 2012 edition [6], and many clinical trials [7, 8] have been carried out to assess the effects of such suggested intervention on patients with severe sepsis and septic shock. However, many intensivists feel there is still lack of effective treatment for severe sepsis and septic shock. It is clear that development of new medication and treatment strategies is urgently needed.

The clinical importance of herbal medicine has drawn substantial attention in recent years. Safflower, which is the dried flower of* Carthamus tinctorius* L., has been used extensively in Chinese medicine for treating gynecological disease and coronary heart disease [9]. Safflower yellow is the main effective component derived from* Carthamus tinctorius* L., and it has been reported to exhibit anticoagulative, vasodilatory, antioxidative, and anti-inflammatory effects [10–16]. In patients with severe sepsis and septic shock, phenomena such as activation of the coagulation system, hypercoagulability of the blood, and release of inflammatory mediators and cytokines, as well as adhesion and aggregation of neutrophils, are commonly found [17, 18]. However, safflower yellow has not previously been employed for treatment of severe sepsis or septic shock. According to the pharmacologic effects of safflower yellow noted above, we hypothesized that intervention with safflower yellow may decrease the mortality in patients with severe sepsis and septic shock. Here, for the first time, a prospective randomized controlled trial was conducted on patients with severe sepsis and septic shock in a poverty-stricken area in western China.

## 2. Methods

### 2.1. Approval of Study Design

This prospective randomized controlled study was approved by the Medical Ethics Committee (number 2012-A-1) of the People's Hospital of Pujiang County, Sichuan Province, China. This study is registered with http://www.chictr.org.cn/ (ref. ChiCTR-TRC-14005196).

### 2.2. Eligibility

Eligible adult patients signed informed consent forms before they were enrolled in this randomized controlled trial. Written informed consent was obtained from all participants. Inclusion criteria included patient age of 18 to 85 years, with a diagnosis of severe sepsis or septic shock according to the diagnostic standards of the 2012 severe sepsis and septic shock treatment international guidelines. Exclusion criteria were as follows: (1) hypovolemic shock, cardiogenic shock, distributive shock, or obstructive shock; (2) pregnancy or lactation in female patients; (3) patient allergy to safflower yellow; (4) current patient enrollment in other medical research; (5) severe disease of the liver and/or kidney.

All participants were informed about the two methods to be used in this trial including conventional therapy according to the international guidelines for management of severe sepsis and septic shock 2012 (control group) and conventional therapy plus safflower yellow treatment (study group).

### 2.3. Treatment

All patients were assigned to either the study group or the control group according to a number extracted at the beginning of the study from a table of random numbers generated by the Statistical Package for the Social Sciences (SPSS) version 17.0 (SPSS Inc., Chicago, IL, USA). This was done only once, and there was no subsequent modification of number assignment during the trial. The study group assignments were placed in sealed, opaque, randomly assorted envelopes. The envelope was not opened until the patient was enrolled in the trial. Patients and statisticians were both blinded to the use of safflower yellow.

Patients in the two groups received 3-hour and 6-hour bundles of conventional therapy according to the international guidelines for management of severe sepsis and septic shock 2012 [9]. Patients in the study group received intravenous injection of safflower yellow at a dose of 100 mg [19] every 12 hours for 72 hours in addition to therapy.

According to Surviving Sepsis Campaign (SSC) international guidelines for management of severe sepsis and septic shock 2012, we collected samples for the culture isolation of the pathogen in each case within 1 hour after patient's arrival in the ICU, or within 3 hours after arrival in the emergency department.

### 2.4. Pharmacology and History of Safflower Yellow

**Figure 1:**
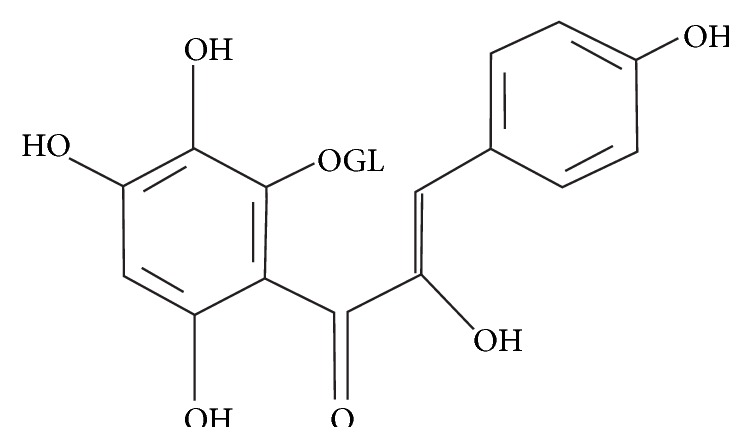
The structure of safflower yellow.

Safflower yellow [20] is the main effective constituent of Flos Carthami. The molecular structure is shown in Figure 1 [20]; it has a molecular weight of 612.53, and the chemical formula is C_27_H_32_O_16_ [20]. The major chemical ingredient of the safflower yellow injectable extract used in this study was hydroxyl safflower yellow A (HSYA) or safflomin A. Flos Carthami, a traditional Chinese herbal medicine, was extensively employed to deal with menstrual problems, cardiovascular disease, pain, and swelling associated with trauma [21]. Flos Carthami is the flower of* Carthamus tinctorius* Linn., a diploid oilseed crop which has been domesticated in the Fertile Crescent region over 4,000 years ago. Full botanical plant name is* Carthamus tinctorius* Linn., Asteraceae [22, 23].

### 2.5. Outcomes

The 28-day mortality from all causes and in-hospital mortality, as well as Kaplan-Meier survival curves, were evaluated in the two groups as primary outcomes.

Secondary outcomes included patient respiratory frequency (*F*), heart rate (HR), urine output, blood pressure, arterial partial pressure of oxygen (PaO_2_), lactate level, bilirubin level, and serum creatinine level, which were measured and assessed every 12 hours for 72 hours. Arterial blood gas values, lactate concentrations, coagulation-related variables, and clinical variables required for determination of the Acute Physiology and Chronic Health Evaluation (APACHE II) score (on a scale from 0 to 71, with higher scores indicating more severe organ dysfunction) were obtained at baseline (0 hour) and at 72 hours. Patients were followed up clinically for 28 days. The number of days of ICU hospitalization, length of time on mechanical ventilation, and renouncement rate of voluntary patient withdrawal from treatment in the two groups were also examined.

### 2.6. Statistical Analysis

In order to initially calculate the sample size required for this study, we first posited that the mortality of the study group with safflower yellow intervention would be 30% lower than the control group. We used data from a previous septic sepsis trial with 52.5% mortality in the control group [24]. Assuming a rate of patient withdrawal from the trial of 20%, to achieve a two-sided type I error rate of 5% and a power of 80%, we calculated that a sample size of 100 patients was required to detect differences in mortality between these two groups. Numbers (%) for categorical variables were compared using Pearson chi-square test or Fisher's exact test. Normally distributed continuous variables were presented as mean ± standard deviation. Statistical significance was determined by Pearson chi-square test, Student's *t*-test, Wilcoxon Rank-Sum Test, and Log Rank Kaplan-Meier analyses. *P* values less than 0.05 were considered to be significant. The Statistical Package for the Social Sciences (SPSS) version 17.0 (SPSS Inc., Chicago, IL, USA) was used for analysis.

## 3. Results

**Figure 2:**
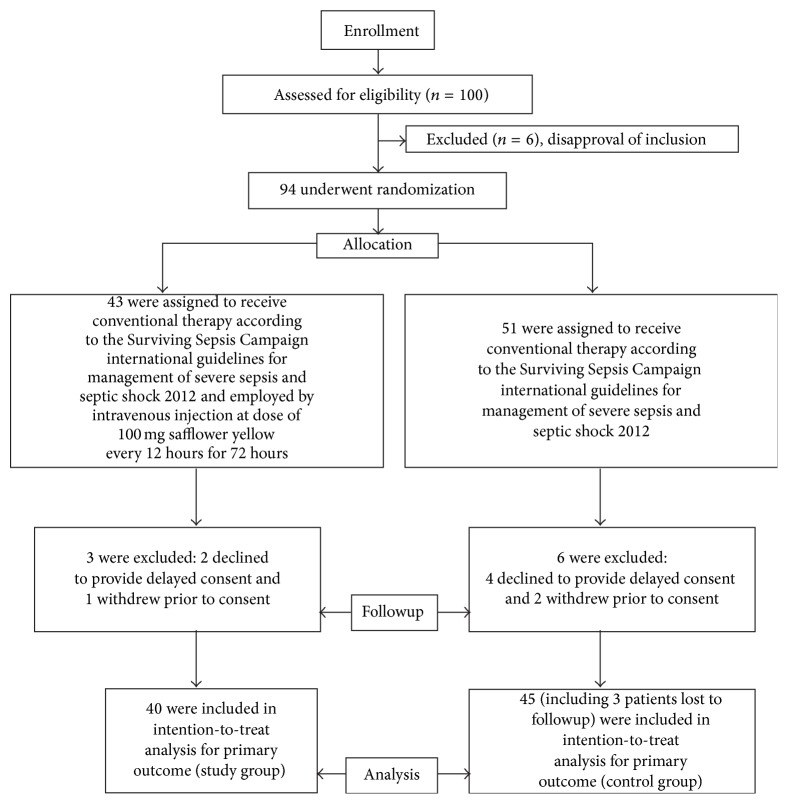
Patients flow diagram.

The trial began on 18 March 2012 and ended on 8 September 2015. It thus lasted almost 41 months. A total of 100 patients were enrolled in this study (Figure 2). Of these patients, 40 were assigned to the study group and 45 were assigned to the control group. All of these 85 patients had medical followup by telephone for 28 days. Three patients were lost to followup in the control group. Thirteen patients in the study group and 10 patients in the control group voluntarily terminated treatment for personal reasons such as financial burdens or poverty in family members. The renouncement rate of voluntary patient self-termination of treatment was not significantly different in the two groups (Table 3).

Basic causes of severe sepsis and septic shock in these patients, critical illness severity scores, and demographic data are summarized for both groups, and both groups showed similar features (Table 1).

**Figure 3:**
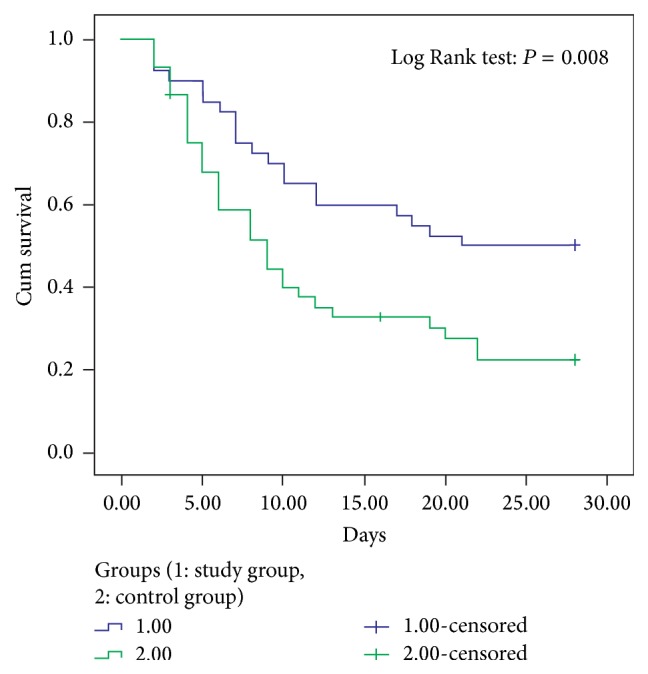
Kaplan-Meier survival curve elevated in study group compared with control group (all causes).

**Figure 4:**
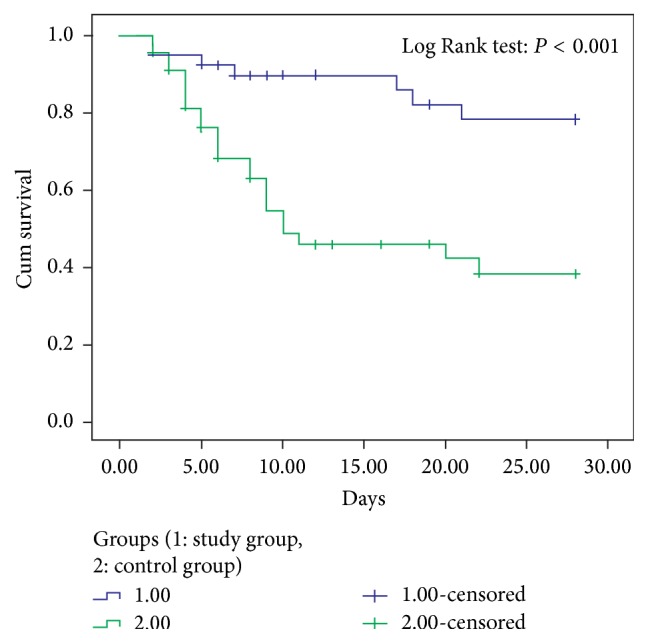
Kaplan-Meier survival curve elevated in study group compared with control group (in-hospital).

After treatment, primary outcomes such as 28-day mortality from all causes and in-hospital mortality (Table 2) were significantly lower in the study group than in the control group (*P* = 0.007 and *P* < 0.001), and the 28-day Kaplan-Meier survival curve was higher in the study group than in the control group (Figures 3 and 4).

Some secondary outcomes, including heart rate, respiratory frequency, leucocyte counts, platelet counts, lactate level, and serum creatinine, decreased, and PaO_2_, mean arterial pressure, and urinary production per hour increased in study group patients as compared to the control group (Table 3). Days of ICU hospitalization and mechanical ventilation showed no significant difference between the two groups (*P* = 0.951, *P* = 0.928) (Table 3).

An allergic reaction was found in 1 patient in the study group, who had erythema, rash, and swelling all over the body. These signs of allergic reaction improved after immediate termination of the safflower yellow infusion and intravenous injection of dexamethasone and calcium glucose. There were no other serious adverse reactions such as allergic shock. Bilirubin levels did not increase after safflower yellow treatment (*P* = 0.844) (Table 3).

Cultures of blood, sputum, abdominal drainage fluid, urine, and pus were performed, with percentages of isolation of 30% and 31.11% in the study and control groups, respectively. These pathogens included* Klebsiella pneumoniae*,* Haemophilus influenzae*,* Escherichia coli*, and* Staphylococcus aureus*. This information about cultured isolation of the pathogens is showed in Table 4.

## 4. Discussion

As we originally hypothesized, this study demonstrated that safflower yellow significantly reduced 28-day mortality and increased survival in patients with severe sepsis and septic shock. Failure of multiple organ systems brings about the high mortality in sepsis and shock, and deterioration of cardiorespiratory function is particularly critical. In this study, we show safflower yellow acts mainly by improving respiratory and cardiovascular function and tissue perfusion, as well as by decreasing inflammatory reaction.

Safflower yellow improved the hemodynamic index of patients with severe sepsis and septic shock as reflected by increases in BP and decreases in the HR and in turn improves the tissue and organ perfusion index. The increase of BP improving tissue perfusion of vital organs was reflected in significant decrease of blood lactate levels compared with those in the control group. Blood lactate levels may be used as an indicator for tissue perfusion during management of severe sepsis and septic shock [25]. In the 3-hour and 6-hour protocols in the guidelines [9], it is necessary to reverse anaerobic metabolism and low tissue perfusion in these patients as quickly as possible. After treatment with safflower yellow, blood lactate levels in the study group decreased significantly, suggesting safflower yellow improves the ischemia hypoxia and anaerobic glycolysis in septic shock.

Previous research reported that administration of esmolol decreased the cardiac workload and safely preserved myocardial function by reducing the heart rate in patients with severe sepsis and septic shock [26]. Similarly, administration of safflower yellow in patients with severe sepsis and septic shock was associated with decreases in the heart rate, which confer benefits such as lengthening of coronary diastolic perfusion time, improvement of coronary perfusion, and alleviation of myocardial ischemia and hypoxia. Consistent with improvement of organ perfusion in, for example, the kidney, urine volume per hour significantly increased with the use of safflower yellow.

Safflower yellow also improved respiratory function. After treatment, respiratory frequency decreased, and respiratory frequency in the safflower yellow group was significantly lower than in the control group. This decrease in respiratory frequency reduced the work of breathing, alleviated respiratory distress, and decreased oxygen consumption. Furthermore, the patients in the study group showed evident increases in PaO_2_ as compared with the control group, representing an increased tissue oxygen supply.

Disordered inflammatory and coagulation function also contribute to the adverse clinical effects in severe sepsis and septic shock. However, safflower yellow improved inflammation indices and coagulation function in these patients. It has been found that safflower yellow effectively inhibits expression of mRNA for proinflammatory factors such as TNF-*α*, IL-1*β*, and IL-6 and promotes expression of anti-inflammatory factors such as IL-10 [27]. Through anti-inflammatory mechanisms, damage to the lung tissue of patients with sepsis and septic shock caused by inflammatory reaction may be reduced, and increases in permeability of blood capillaries may be inhibited. Safflower yellow may also act by blocking the cascade reaction of cytokines that are activated by this disease and thus inhibit activation and adhesion of neutrophils [12, 13, 21]. Leukocyte counts in the study group were significantly decreased as compared with those in the control group after treatment. The platelet counts in the study group were not significantly decreased, but those in the control group decreased significantly, suggesting safflower yellow inhibits platelet activation and aggregation [28].

Serum creatinine was improved in the study group together with urine volume increases as compared with the control group, consistent with safflower yellow acting to improve renal blood flow and perfusion while conferring no injury to renal function. Safflower yellow had no effects on liver function, and after treatment there were no statistically significant differences in bilirubin levels between groups, demonstrating safflower yellow has no hepatotoxicity (Table 3). Employment of safflower yellow thus appears to be safe and effective in the treatment of severe sepsis and septic shock.

During clinical management of patients with severe sepsis and septic shock, safflower yellow improved clinical indices, such as circulation, breath, oxygenation, inflammation response, microcirculation perfusion, and coagulation function, and reduced 28-day mortality and increased 28-day survival. Mortality from all causes was selected as the primary outcome in this study and was as high as 78.58% in the control group and 50% in the study group. In the past, mortality in this disease was very high. The overall mortality from a meta-analysis including 131 studies from 1958 to 1997 was 49.7% [29]. Under current standards of treatment in the developed countries, the overall mortality in the three famous large clinical trials published was 18.70%, 32.14%, and 29.36% at 90 days, respectively [30–32].

There are several possible reasons for this mortality rate from all causes of 78.58% in the control group, which is markedly high as compared with other current studies, as well as the mortality rate from all causes in the study group which is significantly lower than our control group but is still higher than other current studies [30–32]. First, most of the patients in this study were from the countryside and were living in poverty. After an initial period of treatment, if therapeutic intervention did not achieve the level of success expected by the family, or the family could not afford continuing treatment costs, patient family members would give up on all therapy and leave the hospital. Some patient family members from both groups gave up on therapy, including 13 cases in the study group and 10 cases in the control group. All of these 23 patients died within 28 days of returning home, which increased the mortality from all causes. If these 23 patients were excluded, the in-hospital mortality would be 54.76% and 17.5% in the control and study groups, respectively, which is close to other current studies [30–32]. Second, most enrolled patients had severe clinical disease from the outset of treatment, with average APACHE II scores of 29.67 and 30 for the study group and the control group, respectively. Moreover, the score for the sepsis-related organization failure assessment was as high as 12, which is much higher than scores found in similar research [33, 34]. This all argues that these patients had more severe illness and higher mortality risk factors, ultimately giving rise to higher mortality as compared with other studies. Third, this research was conducted in a poverty-stricken area, an agricultural county named Pujiang County, in the west of China. Although we performed this research according to international guidelines for treatment of sepsis and septic shock, the medical conditions, medical facilities, and medical level of the hospital and staff all lagged behind the current international level. However, despite the comparatively high mortality rate in the control group and suboptimal hospital conditions for patient treatment, the critical highlight of this study is that after treatment with safflower yellow in-hospital mortality was reduced to 17.5%, which is closely similar to the mortality reported in studies abroad [35, 36].

This study had some limitations. First, this was a single-center study, and the patients and physicians were from a poverty-stricken area in the west of China. Therefore, the processing of samples was limited. Second, a significant number of family members gave up on therapy after 3 days, influencing the primary outcome (28-day mortality). Third, due to limited conditions, samples were not collected for cellular or molecular experiments to study the molecular mechanisms associated with safflower yellow. Nevertheless, study of the molecular mechanism for safflower yellow in treatment of severe sepsis and septic shock with a larger patient study pool is warranted.

In conclusion, with the use of safflower yellow for therapeutic intervention in severe sepsis and septic shock, the 28-day mortality from all causes and in-hospital mortality were reduced by 28.57% and 37.26%, respectively. Although the rates of mortality for safflower yellow are higher than those reported for conventional treatment in some current studies, they are significantly lower than those for the control group in our study. To our knowledge, this is the first study to evaluate a natural extract of a traditional Chinese herbal medicine for treatment of critical infectious illness, and it is the first time safflower yellow has been used to effectively treat severe sepsis and septic shock. We plan to perform a multicenter RCT clinical trial with a larger number of patients to further study and verify the role and mechanism of safflower yellow in treating severe sepsis and septic shock. However, findings in this study offer a potential new strategy for effectively treating patients with severe sepsis and septic shock.

## Figures and Tables

**Table 1:** Characteristics of the trial patients at baseline.

Demographics	Study group (*n* = 40)	Control group (*n* = 45)	*P* value
Age (years)	64.53 ± 14.89	69.03 ± 12.62	0.189
Weight (kg)	53.90 ± 6.60	53.08 ± 8.75	0.675
Sex (male : female)	28 : 12	28 : 17	0.450
APCAHE II	29.67 ± 7.68	30.39 ± 7.10	0.693
SOFA (sepsis-related organization failure assessment)	12.93 ± 2.46	11.71 ± 2.85	0.190
Comorbidities			
Severe pneumonia	15	13	0.399
Peritonitis	6	11	0.277
Acute exacerbation of chronic obstructive pulmonary disease	10	13	0.549
Severe acute pancreatitis	4	1	0.165
Biliary tract infection	1	3	0.327
Urinary system infection	2	2	1.000
Fracture with infection	1	0	0.488
Multiple organ dysfunction syndrome	1	1	0.741
Burn	0	1	0.512

**Table 2:** Clinical primary outcomes.

Primary outcome measures	Study group (*n* = 40)	Control group (*n* = 42)	Relative risk (95% CI)	*P* value
28-day mortality (all causes)	20/40 (50%)	33/42 (78.58%)	0.636 (0.449–0.901)	0.007
28-day mortality (hospitalization)	7/40 (17.5%)	23/42 (54.76%)	0.320 (0.154–0.661)	<0.001

**Table 3:** Clinical secondary outcomes.

Secondary outcome factors	Study group	Control group	*P* value
	(*n* = 40)	(*n* = 42)	
Heart rate (beats/min)			
Before intervention	122.80 ± 20.89	113.22 ± 27.25	0.106
72 h after intervention	91.63 ± 17.96	112.38 ± 29.13	0.042
Mean artery pressure (mmHg)			
Before intervention	70.43 ± 19.84	74.94 ± 19.25	0.297
72 h after intervention	81.35 ± 15.48	75.02 ± 18.02	0.093
Respiratory frequency (breaths/min)			
Before intervention	25.55 ± 8.19	25.78 ± 6.65	0.282
72 h after intervention	19.87 ± 5.39	23.36 ± 6.05	0.019
Arterial partial pressure of oxygen (PaO_2_, mmHg)			
Before intervention	82.15 ± 45.99	74.51 ± 35.37	0.651
72 h after intervention	107.83 ± 41.51	81.41 ± 39.97	0.015
Leucocyte counts (10^9^/L)			
Before intervention	16.22 ± 8.72	14.82 ± 8.57	0.926
72 h after intervention	12.05 ± 7.75	17.21 ± 8.16	0.013
Urinary production per hour (mL/hour)			
Before intervention	68.28 ± 40.40	74.12 ± 52.99	0.555
72 h after intervention	130.14 ± 93.53	65.7 ± 41.52	0.002
Platelet counts (10^9^/L)			
Before intervention	163.38 ± 120.02	155.83 ± 84.91	0.454
72 h after intervention	156.10 ± 104.79	108.26 ± 64.13	0.018
Lactate level (mmol/L)			
Before intervention	4.68 ± 3.32	4.61 ± 3.55	0.937
72 h after intervention	2.48 ± 2.26	4.40 ± 3.41	0.006
Bilirubin (*µ*mol/L)			
Difference before and 72 h after intervention	13.65 (4.50, 53.10)	17.00 (3.50, 249.63)	0.844
Creatinine (mmol/L)			
Before intervention	137.22 ± 58.62	136.80 ± 65.47	0.976
72 h after intervention	126.52 ± 68.61	160.73 ± 92.27	0.06
Rate of voluntary termination of treatment (%)	32.5% (13/40)	23.80% (10/42)	0.381
Days of ICU hospitalization (days)	8.23 ± 5.25	8.12 ± 10.19	0.951
Days of mechanical ventilation (days)	3.60 ± 4.14	3.70 ± 4.29	0.928

**Table 4:** Culture isolation of the pathogen.

Isolation of the pathogen	Study group (*n* = 40)	Control group (*n* = 45)	*P* value
Positive culture from blood or sterile specimen, number (%)	12 (30%)	14 (31.11%)	0.912
*Klebsiella pneumoniae*	4	3	0.665
*Haemophilus influenzae*	2	3	1
*Escherichia coli*	4	5	1
*Staphylococcus aureus*	1	2	1
Others	1	1	1
